# Efficient Bioflocculation of *Chlorella vulgaris* with a Chitosan and Walnut Protein Extract

**DOI:** 10.3390/biology10050352

**Published:** 2021-04-21

**Authors:** Kaiwei Xu, Xiaotong Zou, Aidyn Mouradov, German Spangenberg, Wenjuan Chang, Yanpeng Li

**Affiliations:** 1 School of Water and Environment, Chang’an University, Xi’an 710054, China; 2018029015@chd.edu.cn (K.X.); 2018029017@chd.edu.cn (X.Z.); 2019129080@chd.edu.cn (W.C.); 2 School of Sciences, RMIT University, Melbourne, VIC 3083, Australia; aidyn.mouradov@rmit.edu.au; 3 Agriculture Victoria, AgriBio, Centre for AgriBioscience, Bundoora, VIC 3083, Australia; German.Spangenberg@agriculture.vic.gov.au; 4 School of Applied Systems Biology, La Trobe University, Bundoora, VIC 3086, Australia; 5 Key Laboratory of Subsurface Hydrology and Ecological Effects in Arid Region, Ministry of Education, Chang’an University, Xi’an 710054, China

**Keywords:** bioflocculation, chitosan, *Chlorella vulgaris*, microalgae, walnut protein extract

## Abstract

**Simple Summary:**

With the increase in population size, global climate changes, and the improvement of living standards, the fossil fuel resources may run out in the future. Microalgae have been considered the next generation of sustainable and renewable feedstock to produce biofuel and a large spectrum of high-value products, such as healthy oils, carotenoids, and proteins. Unlike terrestrial plants, the production of added-value chemicals from microalgal species is not seasonal; they can be grown under climate-independent conditions in bioreactors; can use wastewater as a source of nutrients, contributing to wastewater treatment; and can convert CO_2_ into organic compounds more efficiently. However, the utilization of microalgal biomass is heavily dependent on microalgal biomass harvesting and concentration technology. Flocculation represents a relatively low-cost and efficient approach for the harvesting of microalgal biomass at a large scale. However, in traditional flocculation, most of the chemical flocculants covalently bind to the microalgal surfaces, contaminating the final product, which significantly limits their application. This study aims to develop an efficient and convenient bioflocculation technique to harvest microalgae.

**Abstract:**

Bioflocculation represents an attractive technology for harvesting microalgae with the potential additive effect of flocculants on the production of added-value chemicals. Chitosan, as a cationic polyelectrolyte, is widely used as a non-toxic, biodegradable bioflocculant for many algal species. The high cost of chitosan makes its large-scale application economically challenging, which triggered research on reducing its amount using co-flocculation with other components. In our study, chitosan alone at a concentration 10 mg/L showed up to an 89% flocculation efficiency for *Chlorella vulgaris*. Walnut protein extract (WPE) alone showed a modest level (up to 40%) of flocculation efficiency. The presence of WPE increased chitosan’s flocculation efficiency up to 98% at a reduced concentration of chitosan (6 mg/L). Assessment of co-flocculation efficiency at a broad region of pH showed the maximum harvesting efficiency at a neutral pH. Fourier transform infrared spectroscopy, floc size analysis, and microscopy suggested that the dual flocculation with chitosan and walnut protein is a result of the chemical interaction between the components that form a web-like structure, enhancing the bridging and sweeping ability of chitosan. Co-flocculation of chitosan with walnut protein extract, a low-value leftover from walnut oil production, represents an efficient and relatively cheap system for microalgal harvesting.

## 1. Introduction

Microalgae are considered as the next generation of sustainable and renewable feedstock for the production of biofuel and a large spectrum of high-value products, such as healthy oils, carotenoids, and proteins [1,2,3,4]. Unlike terrestrial plants, the production of added-value chemicals from microalgal species is not seasonal; they can be grown under climate-independent conditions in bioreactors; and they can use wastewater as a source of nutrients, contributing to wastewater treatment [5,6,7,8,9,10]. However, the utilization of algal biomass is heavily dependent on algal biomass harvesting and concentration technology. Usually, the microalgal biomass concentration in an open pond systems and tubular photobioreactors is less than 1 g/L, which is too low to be directly used [11]. Harvesting, as a technology, can contribute for up to 50% of the total cost of production, making the microalgal industry not economically viable [12,13,14]. As a result, this process has become the main bottleneck in the microalgal industry. Nowadays, various harvesting approaches have been applied, including centrifugation, sedimentation, flotation, filtration, and flocculation [8,15,16,17,18,19]. Flocculation represents a relatively low-cost and efficient approach for the harvesting of algal biomass at a large scale [11,20]. Under normal growth conditions, the negatively charged microalgal surfaces prevent their self-flocculation. Flocculation is based on neutralization or reduction of microalgal surface charges using chemical flocculants (inorganic and organic), biological organisms, or using an electrical impulse [14,21,22,23]. Flocculation using positively charged ions was tested with a number of chemicals, such as aluminum sulfate, ferric chloride, polyacrylamide, polyethylene oxide, and others [3,15,21,24,25]. However, most of these chemicals covalently bind to the algal surfaces, contaminating the final product, which significantly limits their application. pH-induced flocculation also has been reported [26,27,28]. The carboxylate ions of the organic matter on microalgal cell surfaces accepted protons when the pH decreases and the negative charges were neutralized, resulting in disruption of the dispersing stability of the microalgal cells. However, the pH decrease-induced flocculation efficiencies for the microalgal cells with a small size (3–5 μm) are low [26]. Bioflocculation using biological organisms, such as bacteria and fungi, attracted attention because of their efficiency and additive effects on biomass production and wastewater treatment [4,10,14,19,29]. Because of the microbial nature of the some of the bioflocculants, these technologies are mainly used in the biofuel industry and for the production of non-food-related products. Application of non-living bioflocculants, including leftovers from the food industry, such as chitosan, starch, and plant and seed components, have been successfully used for the harvesting and concentration of microalgal species [11,30,31,32,33]. A list of their advantages also includes their biocompatibility and biodegradability. Application of cationic starch with a high carbon/nitrogen ratio for harvesting *Chlorella vulgaris* (*C. vulgaris*) led to a 92.6% harvesting efficiency at pH 3 [11]. Teixeira et al. [32] used *Moringa* seed flour to harvest *C. vulgaris,* which showed an 89.0% of flocculation efficiency at pH 9.2. A mung bean protein extract was used as a bioflocculant with a flocculation efficiency of over 92.0% [31].

Chitosan is a product of the deacetylation of chitin, which is the most abundant biopolymer after cellulose [34]. Numerous studies showed that chitosan as a cationic polyelectrolyte could be used as a non-toxic, biodegradable bioflocculant for several algal species [22,30,34,35,36,37,38,39]. Despite its broad application in the bioflocculation of algal cells, the mechanism of chitosan flocculation of algal cells is still not well understood and could vary based on the pH of the media. Flocculation mechanisms of chitosan and its chemical modifications include charge neutralization, charge patching, bridging, and sweeping mechanisms [33]. Chitosan alone and in combination with other chemicals were also widely used for wastewater treatment (for a review, see [33,34,37]). However, the high cost of chitosan (around 20~50 USD kg/L) makes its application for the large-scale flocculation process economically challenging [40]. This triggered research on reducing its amount using co-flocculation with other components [33,40,41,42].

Seed-based proteins were used as coagulants for negatively charged contaminants due to the presence of the abundance of -NH_2_ groups [41]. Walnut (*Juglans regia* L.) is a nutrient-dense food and food supplement that contains a high level of lipids (71% DW) and protein (20% DW). Currently, the largest producer of walnuts, with an annual production of around 1.79 million tons, is China, followed by the USA. The de-fatted biomass, walnut protein extract (WPE), containing essential amino acids and vitamins, has the potential to be used as a source of protein in food supplements used in the baking, meat, and dairy industries [43,44,45]. The proximate composition of WPE is dominated by glutelin (68%) and globulin (24%), with 8% albumin [46]. The amino acid composition of WPE is enriched by aspartic acid and asparagine, glutamic acid and glutamine, and arginine, which meets the requirement of all the essential amino acids for an adult human [46]. However, due to the high presence of polyphenols and condensed tannins, which bind covalently to proteins, the solubility of WPE is low [47,48]. This makes WPE a low-value leftover from the production of valuable walnut oil, typically used as fertilizer or discarded [45,49,50].

This study aims to develop an efficient and convenient method to harvest microalgae using chitosan and WPE. To our knowledge, this is the first study that report on the use of WPE for flocculation of microalgae. Furthermore, the synergic effect of chitosan and WPE on *C. vulgaris* flocculation performance was evaluated. The effects of key factors, including pH and dosage, were examined. The mechanism of the flocculation process was then investigated by Fourier transform infrared spectroscopy, floc size, and microscopy analysis. Finally, the levels of total lipids, proteins, and carbohydrates in the floc biomass were measured.

## 2. Materials and Methods

### 2.1. Microalgae Strain and Culture Conditions

The freshwater microalgae *C. vulgaris* (FACHB-8) (the Freshwater Algae Culture Collection, Institute of Hydrobiology, Chinese Academy of Sciences, Wuhan, China) was used in this study. It was cultured in 1 L Erlenmeyer flasks using BG 11 medium at 25 ± 3 °C under a light intensity of 100 μmol m^−2^ s^−1^ and photoperiod of 12 h light: 12 h dark for 7 days, and then scaled up with a 1/10 inoculation in a 30 L photobioreactor (PBR, Shanghai Guanyu Biological Technology Co., Ltd., Shanghai, China). The culture was under a stirring rate of 100 rpm and aerated at an air flow rate of 60 L h^−1^, and the pH of the medium was automatically controlled at 7.2 ± 0.3 with 0.1 M HCl and 0.1 M NaOH. Microalgae growth was monitored by recording the cell density values every day. Flocculation experiments were performed when the microalgal culture reached its mid-stationary growth phase.

### 2.2. Extraction of Walnut Protein Extract (WPE)

Walnuts were purchased from the local market at Xi’an, Shaanxi, China. Freeze-dried walnuts were shelled, and the kernels were ground using a mill (Fuwanjia, FWJ-30, Yongkang, China). The protein extract from the walnut kernels was prepared by a modified method according to Sze-Tao and Sathe [48]: Briefly, walnut kernels were defatted using hexane at a ratio of 1:10 (*w*/*v*) for 3 h with constant stirring and then followed by vacuum filtration. The filter cake was extracted with hexane again. This process was repeated three times until the filtrate was clear. After that, the residues were freeze-dried. The obtained dry residues were further ground to 50 mesh flour and stored at −20 °C until further use. The defatted walnut flour was dispersed in deionized water (1:15 *w*/*v*) and then adjusted to pH 9 using a 0.1 M NaOH solution. The slurry was stirred for 1.0 h at room temperature, and then it was centrifuged at 8000× *g* for 10 min at 4 °C to obtain the walnut protein. The total protein content of walnut kernels and the concentration of walnut protein were evaluated using the Lowry method [51].

### 2.3. Flocculation Tests

To assess the flocculation efficiency, 100 mL of the microalgal suspension was stirred at 120 rpm in a 250 mL beaker at pH 7. Chitosan was pretreated according to Zou et al. [52]. The flocculants (chitosan or/and WPE) were added slow into individual test beakers and the pH was adjusted. The microalgal suspensions with flocculants were stirred at 300 rpm for 3 min. The suspensions were allowed to settle for 30 min, and the samples were collected from the middle height of the suspension. Optical density (OD) was measured using a spectrophotometer (UV-2450, Kyoto, Japan) at 680 nm and flocculation efficiency was determined using Equation (1):

(1) $$Flocculation\;efficiency\;\left(\%\right)=\frac{OD_{bf}-OD_{af}}{OD_{bf}-OD_{ac}}\times 100\%$$

where $OD_{bf}$ and $OD_{af}$ are the optical density before and after flocculation, respectively; and $OD_{ac}$ is the optical density of the supernatant after centrifugation of the microalgal suspension.

To assess the kinetics of the flocculation over 60 min, a mixture of *C. vulgaris* and the flocculants was stirred at 300 rpm for 3 min and allowed to settle for 10, 20, 30, 40, 50, and 60 min. The flocculation kinetics were calculated by fitting the data to a first-order kinetics model for individual batch flocculation. The kinetics rate constant *k* was obtained using Equation (2):

(2) $$\ln\left(\frac{F_{m}}{F_{m}-F_{t}}\right)=kt$$

where $F_{t}$ is the flocculation efficiency at time *t*; $F_{m}$ is the maximum flocculation efficiency; *k* is the flocculation kinetics rate constant; and *t* is the flocculation time. 

The harvesting rates of the flocculation tests were plotted against time, in which a linear relation between $\ln\left(\frac{F_{m}}{F_{m}{\;-\;F}_{t}}\right)$ and time *t* was observed. The flocculation kinetics rate constant k can be derived from the slope of the straight line with the correlation factor R^2^ > 0.99, which indicated that the flocculation kinetics followed the first-order model. 

### 2.4. Microscopy, Particle Size Distribution, and Zeta Potential Analysis

Microscopy studies and floc size distribution were conducted using an optical microscope with an attached camera (Olympus CKX31, Tokyo, Japan). Zeta potential was assessed according to Xu et al. [15] using a Delsa Nano C particle analyzer (Beckman Coulter, Pasadena, CA, USA).

### 2.5. Fourier Transform Infrared (FT-IR) Spectroscopy Analysis

The flocculants and *C. vulgaris* were characterized by a FT-IR equipment (IL8CERNGI, PerkinElmer, Waltham, MA, USA) according to the procedure previously described in Xu et al. [15]. 

### 2.6. Biochemical Composition Analyses

Lipid extraction was conducted according to Miranda et al. [53]; the total lipid in each sample was determined by weight difference. Carbohydrate content was measured by the anthrone and sulfuric acid method described in a previous study [54]. 

### 2.7. Statistical Analysis

All analyses were performed in triplicates. The data were presented as the mean ± standard error, and statistical significance was analyzed using one-way analysis of variance (ANOVA) via IBM SPSS Statistics 25 (SPSS Inc., Chicago, IL, USA). Differences were considered significant at *p* < 0.05.

## 3. Results and Discussion

### 3.1. The Effect of Chitosan and WPE on Flocculation of C. vulgaris

Zeta potentials, which show the surface charges of the *C. vulgaris* cells, chitosan, and WPE, are shown in Table 1. As for most of the microalgae, the cell surfaces of *C. vulgaris* are negatively charged at most pH conditions, with −25.3 ± 1.5 mV observed at the neutral conditions (pH 7). The positive charge of the chitosan polymers is gradually decreasing from pH 4 till pH 10, with a strong positive value, 23.9 ± 2.3 mV, observed at pH 7. WPE showed the opposite effect, with an increasing negative charge at a higher pH, showing −12.4 ± 1.8 mV at the neutral conditions.

**Figure 1:**
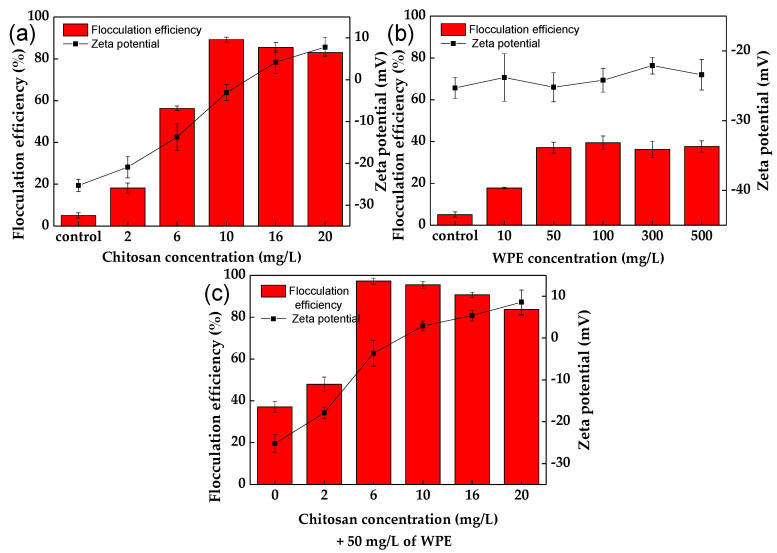
The effect of chitosan, WPE, and chitosan + WPE concentrations on the flocculation efficiency of *C. vulgaris* and the zeta potential of the flocs at pH 7: (**a**) chitosan; (**b**) WPE; (**c**) chitosan + WPE. The error bars represent the standard deviation of the mean (*n* = 3).

The effect of chitosan and WPE on the flocculation of *C. vulgaris* at pH 7 is shown in Figure 1 and Figure S1. Chitosan alone showed a 3.6-fold increase in flocculation of *C. vulgaris*, already at a concentration of 2 mg/L (Figure 1a), with a maximum flocculation efficiency of 89.2 ± 1.2% obtained at 10 mg/L. An increase in the chitosan concentration led to a significant (*p* < 0.05) increase in the zeta potential of the *C. vulgaris*–chitosan floc surfaces, from −25.3 ± 1.5 mV (in control experiments) to 7.8 ± 2.3 mV, as a result of the neutralization of the charges of the *C. vulgaris* cells [34].

The flocculation of different algal cells by chitosan was shown in other studies [23,30,55]. According to the study of Yang et al. [33], the flocculation mechanism of long-chain chitosan leads to destabilization of the algal suspension as a result of the charge neutralization of their surfaces, followed by their aggregation into the large flocs through the bridging effect. According to the study of Cheng et al. [55], chitosan had an isoelectric point around 6.5, and a weak acidic environment made them more efficient as cationic flocculants. However, under a pH lower than 6, the excess of positive charges resulted in re-stabilization of the microalgal cells, decreasing the flocculation efficiency and hindering floc formation [56]. In the study of Xu et al. [23], chitosan of various dosages from 5 to 20 mg/L showed a low flocculation performance (below 10% efficiency) for *Chlorella sorokiniana* at pH 8 and 9.

Negatively charged WPE showed a low effect on flocculation of *C. vulgaris* cells, increasing it 7.3-fold at a concentration of 50 mg/L, with a flocculation efficiency of 37.1 ± 2.6% (Figure 1b). No change in the zeta potential of the microalgal cells was observed after co-cultivation with WPE. According to the study of Rao et al. [57], walnut protein may play a similar role as microalgal organic matter. The presence of glycoproteins creates binding sites, leading to web-like networks with algal cells that enhanced their flocculation [46,57].

Flocs between chitosan and WPE at concentrations of 10 mg/L and 50 mg/L showed positive charges of 12.2 ± 2.2 mV at pH 7 (Table 1). This indicates that their complex could represent a matrix for the flocculation of *C. vulgaris* cells (Figure 1c). Chitosan + WPE showed an additive effect on the flocculation of *C. chlorella* cells, with an efficiency of 97.3 ± 1.4% already at 6 mg/L of chitosan (Figure 1c). The linear structure of the unmodified chitosan creates a bridging effect, which leads to microalgae flocculation [23,33]. The addition of WPE stabilizes chitosan’s web-like structure, which enhances the bridging and sweeping effects in capturing *C. vulgaris*. Kurniawati et al. [58] reported that chitosan can also form complexes with polyanions to increase the microalgae recovery efficiency. 

### 3.2. The Effect of pH on Flocculation of C. vulgaris by Chitosan, WPE, and Chitosan + WPE

**Figure 2:**
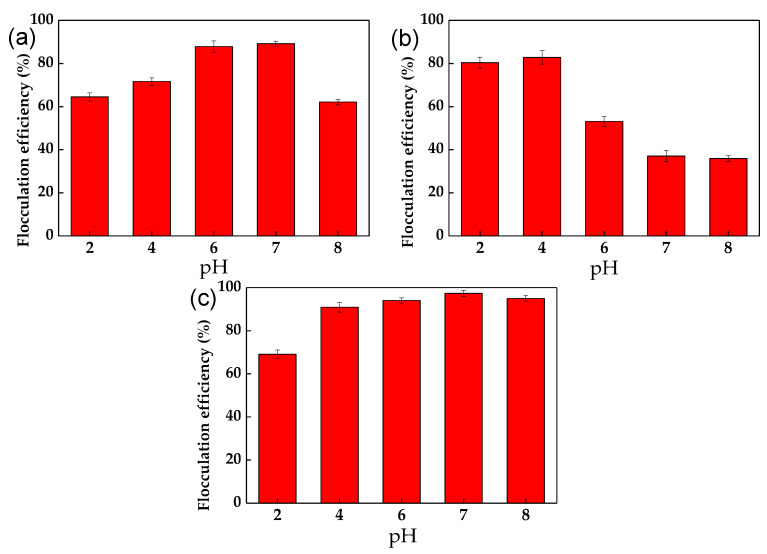
The effect of pH on the flocculation efficiency of *C. vulgaris* by chitosan (**a**); WPE (**b**); and chitosan and WPE (**c**). The error bars represent the standard deviation of the mean (*n* = 3).

The effect of pH on the flocculation efficiency of *C. vulgaris* cells in the presence of chitosan, WPE, and chitosan + WPE is shown in Figure 2. Despite the high positive charges of the chitosan molecules at low pH, the flocculation of the *C. vulgaris* cells was higher at pH 6–7 (Table 1, Figure 2a). Higher pH conditions also showed a reduced level of algal cell flocculation by WPE, which showed a dramatic reduction in flocculation efficiency (Table 1, Figure 2b).

Mixing chitosan with WPE at different pH values followed the pattern of the chitosan charges, but at lower values because of the neutralization of chitosan by positively charged WPE (Table 1). As a result, chitosan + WPE showed a high flocculation efficiency under broad pH conditions, from pH 4 to pH 8 (Figure 2c).

Our results are similar to those of the bioflocculation of *Nannochloropsis* spp. with mung bean protein extract, with a flocculation efficiency 81.0% at pH 2 after a 30 min setting time [31]. However, this system showed a poor flocculation performance (below 20% efficiency) at a neutral pH. Similar to our study, Kong et al. and Mao et al. [46,59] showed that WPE has a low solubility at pH 4, which increases at a pH below 3 or above 5. As a result, precipitated at pH 4, WPE has the highest flocculation performance. In addition, according to the studies of Zou et al. [3] and Yuan et al. [60], at a low pH, chitosan can break into small fragments, which limited its bridging–netting ability. 

### 3.3. Flocculation Kinetics of C. vulgaris by Chitosan and WPE

**Figure 3:**
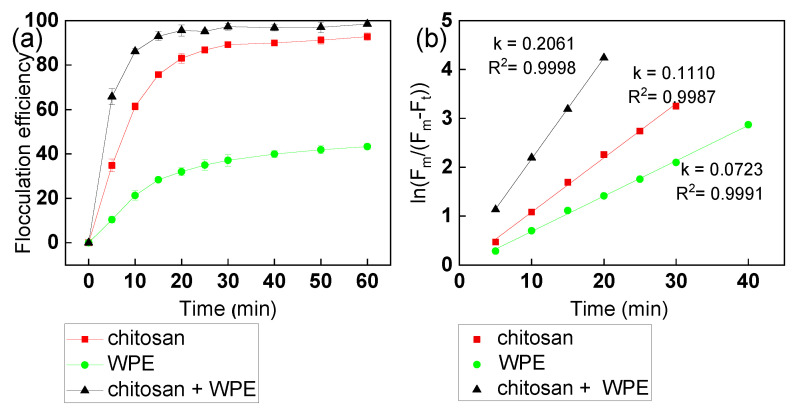
(**a**) The kinetics of *C. vulgaris* flocculation with various flocculants. (**b**) Flocculation kinetic curves of *C. vulgaris* flocculation with various flocculants. The error bars represent the standard deviation of the mean (*n* = 3).

The kinetics of flocculation of *C. vulgaris* with chitosan and WPE at a pH 7 over 60 min is shown in Figure 3. As predicted, WPE alone showed modest flocculation kinetics, reaching just 40% flocculation after 1 hour of incubation. Chitosan showed up to 80% flocculation of *C. vulgaris* cells after the first 20 min. The addition of WPE increased the flocculation efficiency up to 95% over the same period (Figure 3a). The flocculation equilibrium time was about 20 min for chitosan + WPE, 30 min for chitosan, and 40 min for WPE, respectively. The kinetic rate constants were flocculants dependent: 0.111 min^−1^ for chitosan, 0.072 min^−1^ for walnut protein, and 0.206 min^−1^ for chitosan and walnut protein, respectively (Figure 3b). This suggested that chitosan + WPE had a maximal adsorption rate for *C. vulgaris* in the flocculation process, saving flocculation time for harvesting *C. vulgaris*. 

Other protein extracts, such as the *Moringa oleifera* protein used as a bioflocculant, showed a 78% harvesting efficiency for *Chlorella* spp. [61]. Li et al. [62] reported an 87.9% flocculation efficiency using a protein extract from *Shinella albus* to harvest *C. vulgaris*. Additionally, according to Divakaran and Pillai [30], good flocculation of chitosan (up to 90%) was achieved within a narrow pH range (approximately 6 to 8). However, Vu et al. [40] reported that the flocculation efficiency of chitosan at pH 8.05 was not only much lower (below 65%), but it also required a dose twenty times more than that of the synthetic cationic polymer to achieve the same flocculation efficiency around 60%. The addition of inorganic salts, ferric chloride, and aluminum sulfate led to a significantly higher flocculation efficiency, 81% and 89%, respectively. The flocculating performances of chitosan and WPE compared with other flocculants are shown in Table S1.

### 3.4. Size Distributions of C. vulgaris Flocs with Chitosan and WPE

**Figure 4:**
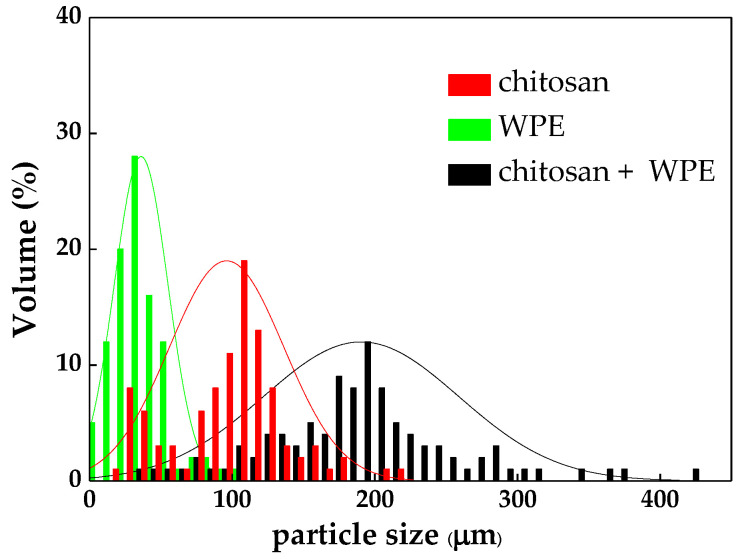
Size distributions of the *C. vulgaris* flocs with chitosan and WPE.

The size of the algal flocs during flocculation is crucial in flocculation efficiency since it determines their settling rate, properties of the flocs, and flocculation efficiency [22]. The floc size can be significantly impacted by several physical and chemical types of interactions between the components, which include charge neutralization, bridging, and sweeping [21]. Figure 4 shows the size distributions of the *C. vulgaris* flocs formed by chitosan, WPE, and chitosan + WPE. The average diameter of the flocs formed by WPE, chitosan, and chitosan + WPE were 36.32 μm, 96.39 μm, and 192.03 μm, respectively.

**Figure 5:**
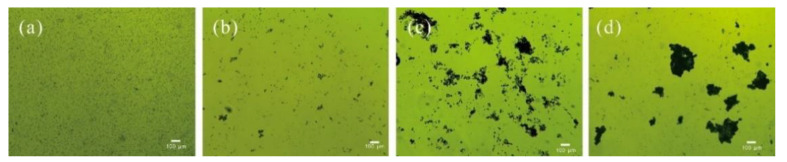
Microscopic images of *C. vulgaris* (**a**), and *C. vulgaris* flocs with WPE (**b**), chitosan (**c**), and chitosan and WPE (**d**).

The detailed microscopic analysis showed that the flocs formed by chitosan and WPE alone were fluffy and unstable in water (Figure 5). Flocs formed by *C. vulgaris* with chitosan + WPE were not only larger in size but also more compressed. It was reported that the structure of the flocs caused by chitosan was thin and fluffy due to a single straight chains of chitosan molecules [33,63].

**Figure 6:**
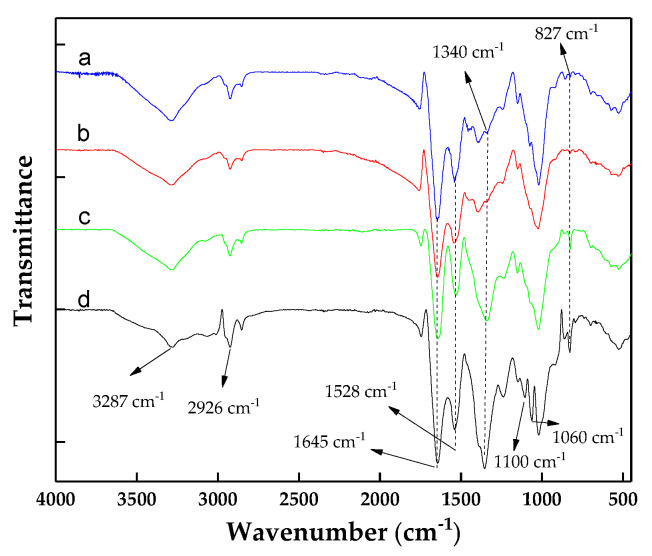
The FT-IR spectra of *C. vulgaris* and the *C. vulgaris* flocs: (**a**) *C. vulgaris*, (**b**) *C. vulgaris* flocs formed by chitosan, (**c**) *C. vulgaris* flocs formed by walnut protein, and (**d**) *C. vulgaris* flocs formed by chitosan and walnut protein.

To assess the possibility of a chemical interaction between *C. vulgaris*, chitosan, and WPT during flocculation, FT-IR spectra of all the components were analyzed (Figure 6). Similar to Yuan et al. [57] and Zou et al. [52], *C. vulgaris* showed a broad absorption band around 3287 cm^−1^, which corresponded to the overlap of the O–H and N–H stretching vibration, and the peak at 2926 cm^−1^ attributed to the stretching of O–H and C–H. The sharp peak at 1645 cm^−1^ referred to the primary amide group (N–H stretching) and imine group (C=N stretching). The peaks at 1528 cm^−1^, 1391 cm^−1^, and 1340 cm^−1^ belong to the nitro group (N–O stretching), the carboxylic acid (O–H bending), and an aromatic amine group (C–N stretching). The amine group, acetal, and alkene were associated with peaks at 1243 and 1020, 1176, 1020, and 827 cm^−1^, respectively. Chitosan alone did not change the FT-IR spectrum of *C. vulgaris*, suggesting the lack of a chemical reaction between these molecules and the surface of the algal cells; thus, the crucial role of electrostatic interactions in this type of flocculation is highlighted. The spectrums of the *C. vulgaris* + WPE flocs showed a difference in absorption intensity between 1340 and 827 cm^−1^, suggesting a chemical interaction between these components. The spectrum of *C. vulgaris* flocs formed by chitosan + WPE showed two new bands, 1100 cm^−1^ and 1060 cm^−1^, which were missing in the *C. vulgaris* + WPE flocs. The appearance of these new peaks suggests a chemical interaction between the *C. vulgaris* + WPE + chitosan flocs. The effect of chitosan and WPE on the flocculation of *C. vulgaris* can be triggered by the interaction between the amino groups of the walnut protein and chitosan chain, forming a web-like structure, as shown in Figure S1.

### 3.5. Biochemical Composition of the WPE and Floc Biomass

**Figure 7:**
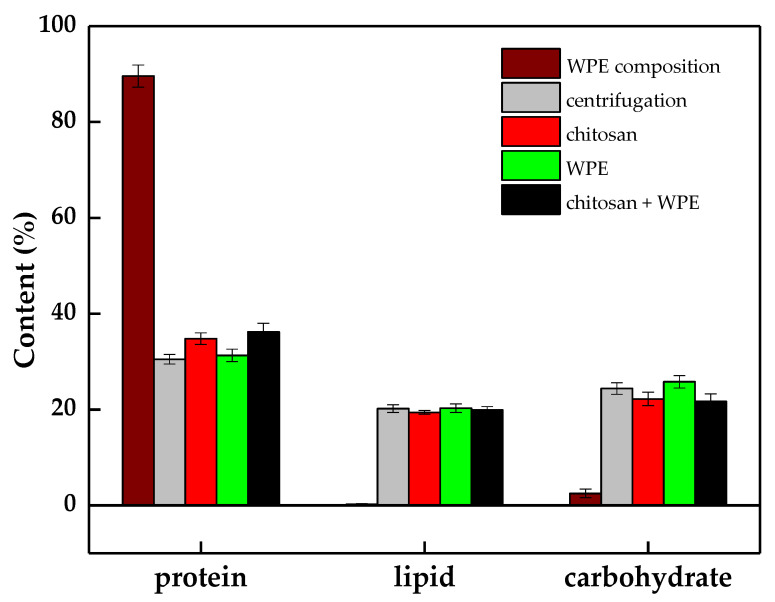
WPE composition extractions (protein, lipid, and carbohydrate) of microalgal cells harvested by direct centrifugation and different flocculants. The error bars represent the standard deviation of the mean (*n* = 3).

The total protein content in the dehulled walnut kernel was 16.5 ± 0.3%, which was closed to the value (19.84 ± 0.22%) reported by Kong et al. [46]. The levels of total lipids, proteins, and carbohydrates in the floc biomass are shown in Figure 7. The total protein content of the WPE, chitosan, and *C. vulgaris* were 89.6 ± 2.3% DW, <2.0% DW, and 30.5 ± 1.0% DW, respectively. As a result of the bioflocculation, in all floc biomasses, the total protein content was found to be about 31.3 ± 1.3–36.2 ± 1.8% DW, which indicates that the contribution of WPE to the final biomass was not significant. Similar to the protein content, the concentration of total lipids and carbohydrates in the flocs were mainly represented by their levels in the harvested algal biomass under an 89.2% harvesting efficiency. This can be explained by the minor ratio DW of the WPE and chitosan in the collected floc biomass. The original amounts of WPE and chitosan added to the algal suspension were just 3.8% and 0.9% of the collected biomass.

Similar to our study, Zhu et al. [64] and Gupta et al. [65] found that there were no significant differences in lipid content in the collected microalgal biomass harvested by chitosan. Moreover, they found a decrease in the lipid content after harvesting by metal ionic flocculants compared to centrifugation.

## 4. Conclusions

In this study, WPE was first used as a flocculant to harvest microalgae, and we developed a dual flocculation system of chitosan and WPE, which can achieve an efficient and rapid flocculation. Results suggested that chitosan and WPE can form a web-like structure, which enhanced the bridging and sweeping ability of chitosan. In addition, the floc biomasses showed an additive composition of proteins, carbohydrates, and lipids in their components. Therefore, the dual flocculation system of chitosan and WPE has a great potential for harvesting microalgae.

## Figures and Tables

**Table 1:** The effect of pH on the zeta potential of *C. vulgaris*, chitosan, WPE, and their flocs. The data are shown as the mean ± standard error (*n* = 3).

Components	Zeta Potential (mV)					
	pH 2	pH 4	pH 6	pH 7	pH 8	pH 10
*C. vulgaris*	5.1 ± 0.4	−10.2 ± 1.2	−17.6 ± 1.0	−25.3 ± 1.5	−23.4 ± 1.7	−25.0 ± 2.4
Chitosan	27.7 ± 3.8	31.6 ± 4.4	29.7 ± 3.3	23.9 ± 2.3	15.6 ± 3.2	8.4 ± 2.6
WPE	7.3 ± 2.5	−1.3 ± 1.4	−11.1 ± 4.2	−12.4 ± 1.8	−17.6 ± 2.4	−20.7 ± 3.5
WPE + chitosan	22.7 ± 4.3	23.8 ± 2.4	21.5 ± 2.7	12.2 ± 2.2	9.6 ± 1.8	1.6 ± 4.2
*C. vulgaris* + chitosan	11.3 ± 1.7	8.6 ± 2.3	6.4 ± 2.0	−3.1 ± 1.9	−5.3 ± 0.9	−15.7 ± 2.8
*C. vulgaris* + WPE	4.7 ± 1.5	−7.8 ± 2.8	−15.4 ± 2.7	−25.2 ± 2.1	−26.5 ± 1.2	−25.6 ± 1.2
*C. vulgaris* + chitosan + WPE	9.5 ± 3.8	3.9 ± 3.4	0.4 ± 1.8	−3.6 ± 3.1	−3.9 ± 2.7	−12.2 ± 3.3

## Data Availability

Not applicable.

## Supplementary Materials

The following are available online at https://www.mdpi.com/article/10.3390/biology10050352/s1. Table S1: Current and reported harvesting methods using bio-flocculants for respective microalgae species; Figure S1: Schematic diagram of flocculation process.
