# Supplementary material for: Efficient Bioflocculation of Chlorella vulgaris with a Chitosan and Walnut Protein Extract
Source: Biology (Basel). 2021 Apr 21;10(5):352. doi: 10.3390/biology10050352 (PMC8143315; doi:10.3390/biology10050352)
Supplement: Supplementary file 1 [file biology-10-00352-s001.zip › biology-1171849-supplementary.pdf]

biology

MDPI

Supplementary

Efficient Bioflocculation of *Chlorella vulgaris* with Chitosan and Walnut Protein Extract

Kaiwei Xu, Xiaotong Zou, Aidyn Mouradov, German Spangenberg, Wenjuan Chang, Yanpeng Li

Table S1. Current and reported harvesting methods using bio-flocculants for respective microalgae species.

Species	Flocculant	Flocculant dosage	pH condition	Flocculation efficiency (%)	Reference
Chlorella vulgaris	Chitosan and walnut protein	6 mg/L of chitosan and 50 mg/L of walnut protein	7	97.3	Current study
Chlorella vulgaris	Chitosan	10 mg/L	7	89.2	Current study
Chlorella vulgaris	Walnut protein	50 mg/L	4	82.8	Current study
Nannochloropsis sp.	Mung bean protein extract	20 mL/L	2	> 90	[1]
Chlorella sp.	Moringa seed protein	10 mg/L	6.5	78	[2]
Chlorella vulgaris	Supernatant of *Shinella albus* xn-1 culture	43.33 mg/L	10.72	89.09	[3]
Chlorella vulgaris	Chitosan and ferric chloride	50 mg/L of chitosan and 40 mg/L of ferric chloride	8.05	81	[4]
Chlorella vulgaris	Chitosan and aluminum sulphate	80 mg/L of chitosan and 40 mg/L of ferric chloride	8.05	89	[4]

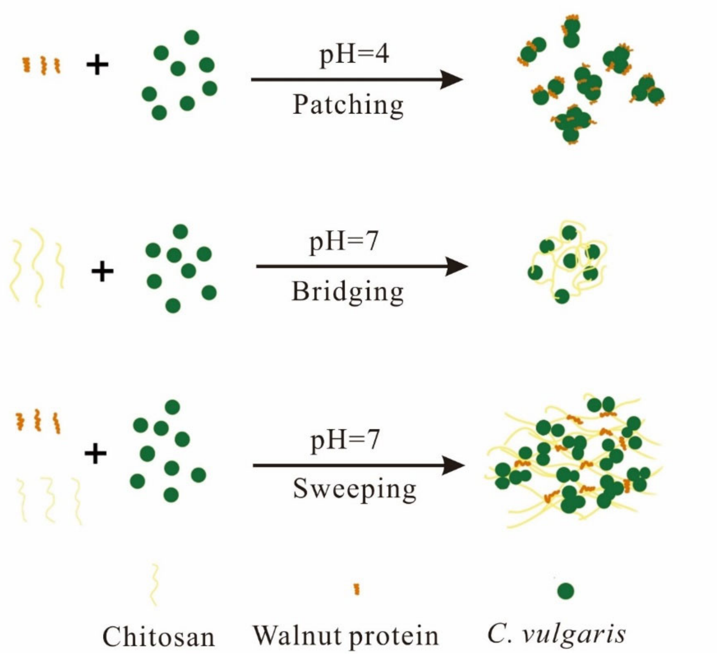

Figure S1. Schematic diagram of flocculation process.

References

1 G. Kandasamy, S.R.M. Shaleh, Harvesting of the microalga Nannochloropsis sp. by bioflocculation with mung bean protein extract, Applied biochemistry and biotechnology, **2017**, *182*, 586-597.

2 S.H.A. Hamid, F. Lananan, H. Khatoon, A. Jusoh, A. Endut, A study of coagulating protein of Moringa oleifera in microalgae bio-flocculation, International Biodeterioration & Biodegradation, **2016**, *113*, 310-317.

3 Y. Li, Y. Xu, L. Liu, X. Jiang, K. Zhang, T. Zheng, H. Wang, First evidence of bioflocculant from Shinella albus with flocculation activity on harvesting of Chlorella vulgaris biomass, Bioresource Technology, **2016**, *218*, 807-815.

4 H.P. Vu, L.N. Nguyen, G. Lesage, L.D. Nghiem, Synergistic effect of dual flocculation between inorganic salts and chitosan on harvesting microalgae Chlorella vulgaris, Environmental Technology & Innovation, **2020**, 17, 100622.
